# 10 years of BAWLing into affective and aesthetic processes in reading: what are the echoes?

**DOI:** 10.3389/fpsyg.2015.00714

**Published:** 2015-06-03

**Authors:** Arthur M. Jacobs, Melissa L.-H. Võ, Benny B. Briesemeister, Markus Conrad, Markus J. Hofmann, Lars Kuchinke, Jana Lüdtke, Mario Braun

**Affiliations:** ^1^Department of Experimental and Neurocognitive Psychology, Freie Universität BerlinBerlin, Germany; ^2^Cluster of Excellence “Languages of Emotion”, Freie Universität BerlinBerlin, Germany; ^3^Dahlem Institute for Neuroimaging of EmotionBerlin, Germany; ^4^Scene Grammar Lab, Department of Cognitive Psychology, Goethe University FrankfurtFrankfurt, Germany; ^5^Department of Cognitive, Social and Organizational Psychology, Universidad de La LagunaSan Cristóbal de La Laguna, Spain; ^6^Department of Psychology, General and Biological Psychology, University of WuppertalWuppertal, Germany; ^7^Experimental Psychology and Methods, Faculty of Psychology, Ruhr Universität BochumBochum, Germany; ^8^Centre for Cognitive Neuroscience, Universität SalzburgSalzburg, Austria

**Keywords:** Berlin Affective Word List (BAWL), valence decision task, lexical decision task, emotion, word recognition models, neurocognitive poetics, reading, aesthetics

## Abstract

Reading is not only “cold” information processing, but involves affective and aesthetic processes that go far beyond what current models of word recognition, sentence processing, or text comprehension can explain. To investigate such “hot” reading processes, standardized instruments that quantify both psycholinguistic and emotional variables at the sublexical, lexical, inter-, and supralexical levels (e.g., phonological iconicity, word valence, arousal-span, or passage suspense) are necessary. One such instrument, the Berlin Affective Word List (BAWL) has been used in over 50 published studies demonstrating effects of lexical emotional variables on all relevant processing levels (experiential, behavioral, neuronal). In this paper, we first present new data from several BAWL studies. Together, these studies examine various views on affective effects in reading arising from dimensional (e.g., *valence*) and discrete emotion features (e.g., *happiness*), or embodied cognition features like *smelling*. Second, we extend our investigation of the complex issue of affective word processing to words characterized by a mixture of affects. These words entail positive and negative valence, and/or features making them beautiful or ugly. Finally, we discuss tentative neurocognitive models of affective word processing in the light of the present results, raising new issues for future studies.

## Introduction

The aim of this paper is to discuss the contribution of a lexical data-base, the BAWL, to the study of affective and aesthetic processes in reading. We start with a short overview of studies using the BAWL in a variety of experimental settings that investigate a wide range of questions covering perceptual-attentional, memory, affective-aesthetic, or social-emotional issues. We then present a re-analysis of the original BAWL data (Võ et al., 2006) suggesting that both discrete emotion and embodiment or semantic richness variables also affect processing of the BAWL words. Results of three new studies from our lab investigating affective lexical semantics are subsequently discussed: one uses a special version of the BAWL to look at affective lexical semantics in children, one uses a novel class of stimuli that have a clear bivalent affective semantic structure, and the last one looks at what makes words beautiful or ugly. The paper ends with a discussion of tentative neurocognitive models of affective word recognition in the light of results from this and other recent publications addressing the *How, Where*, and *When* questions of valence ratings and decisions.

Experimental research on visual word recognition and reading has long neglected the fact that those high-dimensional symbolic stimuli called words have properties relating to our bodily sensations and actions, as well as to our affective system. Thus, popular models of visual word recognition, text processing, or reading remained completely silent with regard to potential affective or aesthetic effects of words (Jacobs, 2011). This might come as a surprise considering that early theoreticians of language, such as Freud (1891) or Bühler (1934), already argued that both spoken and written words are embodied stimuli with the potential to elicit overt and covert sensory-motor and affective responses. For example, Bühler introduced the notion of “Sphärengeruch” (spheric fragrance of words), according to which words have a substance, and the actions they serve - speaking, reading, thinking, feeling – are themselves substance-controlled. He gives the example of the word “Radieschen” (*garden radish*) that can evoke red or white color impressions, crackling sounds, or earthy smells and spicy tastes in the minds of readers and transport them either into a garden or to a dinner table, which creates an entirely different “sphere” as, say, the word “ocean”. The renaissance of Bühler's ideas in recent theories of symbol grounding, embodied cognition, or neural re-use (Niedenthal, 2007; Anderson, 2010; Willems and Casasanto, 2011) can explain why evolutionary young cultural objects like words can evoke basic and fiction emotions as well as aesthetic feelings at the subjective-experiential level of observation, and also activate affective processing networks at the neuronal level. As outlined in Schrott and Jacobs (2011) the challenge here is to bridge the gap between neurobiological theories of emotion, as perhaps best represented by Panksepp's (1998) core affect systems theory, and complex (psycho-)linguistic models, as exemplified by Jakobson's (1960) extended version of Bühler's (1934) ‘organon model’ of language functions. Since evolution had no time to invent a proper affective system for art reception, even less so for reading, the emotional and aesthetic processes we experience when reading must be somehow linked to the ancient neuronal affect circuits we share with all mammals. As a concise name for the latter assumption about the emotion-language link we have coined the term ‘Panksepp-Jakobson hypothesis’ (Jacobs and Schrott, 2013; Jacobs, 2015b), which finds indirect or direct support in many papers from our lab and others (e.g., Cupchik, 1994; Kneepkens and Zwaan, 1994; Miall and Kuiken, 1994; Oatley, 1994; Kuchinke et al., 2005; Kissler et al., 2007; Hofmann et al., 2009; Schacht and Sommer, 2009; Briesemeister et al., 2011a,b; Altmann et al., 2012, 2014; Bohrn et al., 2012a,b, 2013; Briesemeister et al., 2012, 2014a,b; Ponz et al., 2013; Hofmann and Jacobs, 2014; Hsu et al., 2014; Jacobs, 2014a,b; Hsu et al., 2015a,b,c).

## The “Berlin Affective Word List” (BAWL) as a basic tool for studying affective and aesthetic processes in reading

Limbach's (2004) wonderful book presenting the results of the election of the most beautiful German words over many years, makes readers discover impressive examples for the fact that even 9-year old children can find discrete emotions, such as joy, or feelings of beauty in single words and can also convincingly argue why (Schrott and Jacobs, 2011). These examples leave no doubt that words can be positive or negative, beautiful or ugly, more or less exciting or calming, evoke mental images of sensory-motor events, or feelings of happiness. They also support the notion of *one-word poetry*, i.e., that single utterances or words—even outside lyrical contexts—can fulfill what Jakobson called *the poetic function* and cause aesthetic emotions (Jakobson, 1960; Jacobs and Kinder, 2015).

However, introspections and intuitions about how words can evoke affective and aesthetic processes are one thing; experimentally demonstrating this is yet another. Here we will not immerse into discussions on what emotions are (Kagan, 2010). Rather, we focus on the empirical demonstration of different word properties and their influence on recognition processes that can be meaningfully related to theories of emotion covering a wide spectrum from the classical valence/pleasantness and arousal/activation dimensions of words, to discrete emotion and embodied cognition features, as estimated by ratings of joy/happiness, disgust, or smelling.

To provide a basic tool for researchers interested in affective reading processes in the German language, we have over the last 10 years developed the BAWL—providing valence, arousal, and imageability ratings for approximately 3000 German words. Table 1 summarizes more than 50 studies (until November 2014) that have used words from the BAWL to study effects of affective word properties (other studies not included here have used the BAWL to *control* for affective word properties, e.g., Briesemeister et al., 2009; Hofmann et al., 2011; Hsu et al., 2014, 2015a,b,c). The majority of these studies used single words and employed an explicit valence decision task (VDT) or an implicit^1^ lexical decision task (LDT). But also various memory tasks with mostly valence as the independent variable (IV) and a variety of dependent variables (DVs) have been used to explore effects on sublexical, lexical, and supralexical levels.

**Table 1:** **Summary of studies using the BAWL for stimulus manipulations**.

**Study**	***N* subjects**	***N* words**	**Task(s)**	**Relevant conditions/IV**	**Relevant method/DV**
Bayer et al., 2011	18	180	Silent reading, LDT, memory	Val Aro	Pupil size
Bayer et al., 2012a	12	180	Reading, LDT	Val Aro	ERPs
Bayer et al., 2012b	25	72	Reading, emotional 1-back task	Val Aro, font size	ERPs
Bayer and Schacht, 2014	25	72	1-back task	Val Aro	Ratings, ERPs
Böttcher and Dreisbach, 2013	38	6	Affective priming VDT	Val	RTs
Briesemeister et al., 2011a	79	1958/175	Discrete emotion ratings, LDT	Disc. Emo	RTs
Briesemeister et al., 2011b	21	125	LDT	Disc. Emo	RTs
Briesemeister et al., 2014a	20	120	LDT	Disc. Emo	RTs, fMRI
Briesemeister et al., 2014b	19	120	LDT	Disc. Emo	RTs, ERPs
Casasanto et al., 2014	–	2902	–	Words	
Conrad et al., 2011	40	240	LDT	Val, L1/L2	RTs, ERPs
Dieler et al., 2010	16	90	Think/no think (suppress) task		fNIRS
Dreisbach and Fischer, 2012	30	6	Affective priming VDT	Prime type	RTs
Eder et al., 2014	41	80	Affective priming	Cue type	RTs
Fritsch and Kuchinke, 2013	21	150	LDT, evaluative conditioning	Val	RTs, ERPs, Hits, FA
Fritz and Dreisbach, 2013	45	112	Affective priming VDT	Prime type	RTs
Fritz and Dreisbach, 2014	88	138	Affective priming VDT	Prime type	% Negative judgments
Frühholz et al., 2011	17	48	Color naming, VDT	Task type, Val, modality	RTs, ERPs
Fuge et al., 2014	541	105	Emotional 2-back task	Task difficulty	Errors
Gärtner and Bajbouj, 2014	20	20	Free recall	Mood type	ERD, ERS
Gole et al., 2012	36	200	Emotional go/no go (VDT)	Trait worry group	RTs, errors
Graupmann et al., 2013	12	120	Liking decision	Prime type	fMRI
Grimm et al., 2012	20	105	Emotional 2-back task	Val Aro	RTs, fMRI
Heister and Kliegl, 2012	40	360	Corpus analysis	Val, corpus type	*r*^2^
Herbert et al., 2013	41	22	Free recall, rating	Eating disorder score	Startle eye blink, heart rate
Hofmann et al., 2009	20	200	LDT	Val Aro	RT, ERPs,sLORETA
Hofmann and Jacobs, 2014		2901		Semantic cohesion	Val
Jansma et al., 2014	14	120	Recognition test	Val	fMRI
Kattner and Ellermeier, 2014	30	6	Free recall	Irrelevant sound (Y/N)	Errors
Klackl et al., 2013	20	96	LDT	Val, words' death-relatedness	RT, ERPs
Kometer et al., 2012	17	?	Emotional go/nogo (VDT)	Val	RT, ERPs
Kopf et al., 2013	30	?	Emotional n-back	Val	fNIRS, ERPs, ERR
Kuchinke et al., 2007	26	180	LDT	Val Freq	RTs, pupil size
Kuchinke and Lux, 2012	66	300	LDT Aro rating	Val Aro Hemisphere Caffeine	Hits, FA
Kuchinke et al., 2013	20	256	Recognition test	Highvs. Low Associates	Hits, FA, fMRI
Kuchinke et al., 2014	21	156	LDT	Val, font familiarity	RTs, ERPs
Kuehnast et al., 2014	815	16	Free association	Words	MDS
Kurtz and Zimprich, 2013	47	80	Several memory tests	Processing speed, verbal knowledge	Verbal learning
Palazova et al., 2011	20	180	LDT	Val Freq wordclass	RTs, ERPs
Recio et al., 2014	29	477	LDT	Val Aro	RTs, ERPs
Rellecke et al., 2011	24	150	Face/word classification task	Val	RTs, ERPs
Schlochtermeier et al., 2013	21	80	VDT	Val	RTs, fMRI
Schnitzspahn et al., 2012	86	195	Prospective memory task	Val	Memory performance
Schwager and Rothermund, 2013	66, 17, 58	36	VDT	Val	RTs
Silveira et al., 2013	32	24	Face attractiveness ratings	Death word priming	fMRI
Tempel et al., 2013	25	120	VDT	Val, stimulus type (words, pictograms)	RTs, ERPs
Võ et al., 2006	21	360	VDT	Val	RT
Võ et al. (2008)	19	180	Old/new recognition decision	Val	RTs,d',C, pupil size
Võ et al. (2009)	200	2900	Val Imag Aro ratings	Words	Ratings
Wabnitz et al., 2012	23	100	Reading	Val, threat words	RTs, ERPs
Wagenbreth et al., 2014	16	192	LDT	Val, emotion category	RTs
Weigand et al., 2013a	15	80	Emoback task	Discrete emo (fear, anger)	tDCS,rTMS
Weigand et al., 2013b	28	60	Emoback task	Discrete emo (fear, anger)	rTMS

*Abbreviations: Val, valence; Aro, arousal; disc. Emo, discrete emotion*.

These studies show that the BAWL is a popular tool for bridging the language—emotion gap in research and that its stimuli are well cross-validated at the three relevant processing levels: *experiential* (e.g., subjective ratings, self-reports; Võ et al., 2006; Schnitzspahn et al., 2012), *behavioral and psychophysiological* (e.g., response times, heart rate, startle reflex, oculo- and pupillometric responses; Kuchinke et al., 2007; Võ et al., 2008; Bayer et al., 2011; Briesemeister et al., 2011a,b; Herbert et al., 2013), and *neuronal* (fMRI, EEG, fNIRS, and TMS or tDCS; Kuchinke et al., 2005, 2006; Hofmann et al., 2009; Conrad et al., 2011; Bayer et al., 2012a,b; Schlochtermeier et al., 2013; Tempel et al., 2013; Weigand et al., 2013a,b; Briesemeister et al., 2014a,b; Gärtner and Bajbouj, 2014; Hsu et al., 2014; Recio et al., 2014), as well as in computational-information technological and cartographic studies (Pak and Paroubek, 2010; Garcia Becerra, 2012; Hauthal and Burghardt, 2014).

Complementing the “Affective Norms for English Words” (ANEW; Bradley and Lang, 1999), the Sussex Affective Word List (SAWL; Citron et al., 2012), or the “Affective Norms for German Sentiment Terms” (ANGST; Schmidtke et al., 2014a), which rely on a dimensional theory of emotion a la Wundt, Lang, or Russell, a recent version of the BAWL, the DENN-BAWL, is also compatible with discrete emotion theories, such as Darwin's or Panksepp's (Briesemeister et al., 2011a, 2014a,b). Even more recent extensions include a multilingual version of the BAWL containing more than 6000 words allowing comparisons between German, Spanish, English, and French (Schmidtke et al., 2014a), and preliminary versions for testing children, the kidBAWL, including embodiment ratings (eBAWL), the noun-noun compound/NNC-BAWL, special versions for clinical applications (cBAWL; Gole et al., 2012; Kometer et al., 2012; Herbert et al., 2013; Gärtner and Bajbouj, 2014), and one for experiments in neuroaesthetics (bBAWL). As shown in the following sections, the BAWL can be used to estimate the emotion potential of lexical or supralexical units, and is complemented at the sublexical level by the EMOPHON tool, allowing to estimate the affective value of sublexical units (Aryani et al., 2013). Together these tools offer the possibility to obtain estimates of the emotion potential and aesthetic aspects not only for single words but also for supralexical units like text passages, poems, or songs, as evidenced by recent studies from our lab (Jacobs et al., 2013; Hsu et al., 2014, 2015a,b,c; Lüdtke et al., 2014; Jacobs, 2015a,b).

## BAWL06 reanalysis of valence decision response times (VDRTs) with a combination of exploratory factor analysis and increasingly complex linear mixed models (LMM)

Researchers interested in affective word properties face the challenge to single out effects of features like valence from more than 50 quantifiable factors known to affect word recognition performance (Graf et al., 2005). Apart from valence and arousal, the about 3000 words validated in our first two BAWL papers (Võ et al., 2006, 2009) are characterized by a dozen relevant psycholinguistic variables, such as word length, neighborhood density or frequency, allowing to disentangle possible affective effects from those factors often confounded with valence or arousal, e.g., imageability (Kousta et al., 2011; Westbury et al., 2013).

In the original paper introducing the BAWL (Võ et al., 2006; henceforth BAWL06), we presented VDRTs as a function of valence ratings for 360 German words and obtained a slightly asymmetric, inverse U-shaped curve, mean RTs being shortest for positive words, followed by negative, and neutral ones. The valence ratings accounted for about 50% of mean RT variance (for a subset of 360 words), thus leaving 50% unaccounted for. Since then, the words in the BAWL have been updated by a number of additional features. So, here we ran a reanalysis of the original data to see which other variables may account for the remaining 50% of variance. Likely candidates are other affective-semantic variables like arousal and imageability, (sub)lexical variables like frequency, number of syllables, or neighborhood density, and discrete emotion variables (Briesemeister et al., 2011a, 2014a,b). Moreover, some recent work has provided evidence that words also possess the potential to evoke bodily sensations and mental imagery associated with the sensory-motor system, one nontrivial source of semantic information (Bühler, 1934; Andrews et al., 2009). We thus also took into account variables related to sensory experience (Juhasz et al., 2011) and body object interaction (Siakaluk et al., 2008), sometimes being considered as parts of a metavariable affecting word recognition called *semantic richness* (Pexman et al., 2008; Yap et al., 2012). In doing so, we followed a mixed approach combining available data for variables such as word frequency or discrete emotion ratings (from the DENN-BAWL) with newly collected ratings of embodiment features. To reduce complexity, the latter were submitted to an exploratory factor analysis which is useful to find possible (latent) factor structures underlying a larger number of variables. This resulted in a tentative three factor solution. A total of 14 variables were then submitted to a stepwise LMM to explore which type (e.g., affective-semantic vs. embodiment) and combination of variables may have played a role in determining the BAWL06 VDRT data (see Appendix in Supplementary Materials for details).

### DENN-BAWL and eBAWL: discrete emotion and embodiment features structure of BAWL06 words

A fine-grained analysis of the 175 words of the BAWL06 for which we had discrete emotion ratings revealed a maximum of 91 (52%) words for which joy/happiness was the “dominant” associated emotion (i.e., maximum rating value of all five discrete emotions), 35 (20%) anger words, 32 (18.5%) fear words, only 10 sad words (6%), and a minimum of six disgust words (3.5%). The “top 3” (i.e., joy rating > 2.5/5) joy words were: SONNE (sun), MEER (sea), and SOMMER (summer), the top anger-related words: STAU (traffic jam), ARROGANZ (arrogance), and GEIZ (avarice), the top fear words: GIFT (poison), UNHEIL (calamity), and MORD (murder), the top sad words: LEID (distress), TRENNUNG (separation), and FRIEDHOF (graveyard), and the top disgust words: GESTANK (stink), ÜBEL (evil), and BAKTERIE (bacteria). However, as a matter of fact, a lot of words do not really have a *dominant* emotion associated with it, but are clearly ambi- or polyvalent, e.g., the word “rocket” (RAKETE) shares a mean joy rating of 1.95 with a mean anger value of 2, and a valence rating of -0.8. Now, is this word *neutral* or *negative*, or does it rather have a mixed affective-semantic structure (Briesemeister et al., 2012)? Other striking examples emphasizing our point are words like SCHLAG (“blow” or “strike”), for which two negative emotions with an opposite approach-avoidance structure compete (average anger value = 2.8, fear value = 2.7), or SCHULD (“guilt”), for which we have a perfectly balanced trivalent structure (anger, sadness, and fear = 2.3).

To examine possible effects of embodied cognition, we collected the following embodiment ratings for about 700 German words (see Appendix A1 in Supplementary Materials), asking to what extent subjects associate a word with seeing/SEE, hearing/HEA, smelling/SME, tasting/TAS, touching/TOU, feeling/sensing/FEE, or moving/MOV (eBAWL, cf. McRae et al., 2005). Looking at the dominant embodiment ratings for all BAWL06 words available (*N* = 193), we found a maximum of 116 (60%) words for which *seeing* was the dominant sensory-motor association, 53 *feeling* words (27%), nine *hearing* words (5%), six *tasting* (3%), five *touch* words (2.5%), three *moving* words (1.5%), and a minimum of only one *smell* word (0.05%). As an illustration, the highest embodiment or E-index (sum of all seven ratings) of all words had: MEER (sea; 31.0), followed by HONIG (honey; 25.9), SCHWESTER (sister; 25.9), and ZIGARRE (cigar; 25.8); the lowest had ZWECK (purpose; 9.8), followed by ZUFALL (chance 10.5), RABATT (discount; 10.7), and SPIONAGE (espionage; 10.9). Similarly to the mixed discrete emotion structure, the BAWL06 words also seem to have a mixed embodied feature structure, as exemplified by words like WAFFE (“weapon”) with very similar ratings for: *touch* (4.88), *seeing* (4.82), and *hearing* (4.53), or TRENNUNG (“separation”): *seeing* (4.76), and *feeling* (4.53).

An exploratory factor analysis (maximum likelihood/varimax) on the seven embodiment variables revealed a significant three-factor structure accounting for about 55% of the variance with acceptable eigenvalues (2.3, 1.4, 1.2). TAS and SME were related to Factor 1 (*Taste*), TOU and SEE to Factor 2 (*Grasp*), MOV and HEA to Factor 3 (*Move*), and FEE only marginally to Factor 3. These three factors were also included in the following analyses.

### Stepwise LMM approach with three affective-semantic, three (sub)lexical, five discrete emotion, and three embodiment variables

The previous analyses indicated that the BAWL06 words have a complex mixed affective-semantic structure that likely contributes to variance in dependent measures such as VDRT or LDRT. We tested this assumption using a stepwise LMM approach whose advantages in psycholinguistic research using two random factors (i.e., participants and words) have been discussed elsewhere (e.g., Baayen et al., 2008; Kliegl et al., 2010; Janssen, 2012; Kuchinke and Lux, 2012; Yap et al., 2012; Lüdtke et al., 2014). Following Janssen (2012), a statistical model of the data using log-transformed VDRT^2^ as dependent variable was built from a null model (two random effects only: participants and words) by stepwise adding all main fixed effects^3^ for three affective-semantic (valence/V, arousal/A, imageability/I), three (sub)lexical (logF, syllables/S, and N), five discrete emotions (joy/happiness/HA, anger/AN, fear/FE, sadness/SA, and disgust/DI), and three embodiment variables (Taste, Grasp, Move). We then started with four simple unmixed models (Affective-Semantic, Lexical, Discrete, Embodiment), after which we entered the eight variables that yielded significant effects in those four models into a complex mixed (CoMi) model using V, A, Syl, HA, AN, SA, Taste, and Grasp as fixed effects (see Table 2). As an additional control model, we tested an LMM combining all 14 variables (14 V model) of the four unmixed models (independently of the significance of their effects). The two best-fitting were also the most complex models (CoMi and 14 V) which could not be discriminated on the basis of the AICc values^4^. A chi-square test using the log-likelihood data (i.e., likelihood ratio test) revealed a significant difference [chi-square (*df* = 6) = 28, *p* < 0.001] favoring the 14 V model.

**Table 2:** **Results of stepwise LMM analysis**.

**Model**	**−2Log likelihood**	**AICc**
Null (two: random effects)	17089	17097
Affective-semantic (three: Val, Aro, Imag)	7423	7437
(Sub)lexical (three: logF, syllables/S, N)	7444	7458
Discrete (five: HA, AN, FE, SA, DI),	8334	8353
Embodiment (three: Taste, Grasp, Move)	9544	9558
Complex Mixed (CoMi) (eight: V, A, Syl, HA, AN, SA, Taste, Grasp)	6967	6992
14 Variables (14 V) (14: 3 affective-semantic, 3 sublexical, 5 discrete, 3 embodiment)	6995	6991

*Number and name of factors in brackets (see Appendix A2 in Supplementary Materials for more details)*.

What emerges from these results is a more complex picture than back in 2006: depending on which variables are entered into LMM or standard multiple regression analyses, VDRTs can be affected by all four groups of variables analyzed here: *affective-semantic, (sub)lexical, discrete emotion*, and *embodiment*. This fits with results from the above mentioned studies showing effects of both discrete emotion and embodiment or semantic richness variables. To what extent those variables interact with each other (and variables not considered here) in influencing simple or transformed RTs from valence/lexical decision or other reading tasks is an issue for future studies. A related question is to what extent rating variables like valence and happiness, arousal and fear, or disgust and smell tap into the same underlying mental/neuronal processes (Westbury et al., 2013, 2014). We believe this issue cannot be decided on the basis of more or less exploratory LMM or regression analyses alone, but requires the research strategy of functional overlap modeling by help of computer models of visual word recognition that have sufficient structure to not only simulate effects of lexical variables, but also of the other three types of factors analyzed here (Jacobs and Grainger, 1994; Grainger and Jacobs, 1996; Hofmann and Jacobs, 2014). We will discuss first steps into this direction at the end of this paper.

## Affective lexical semantics in children: the kidBAWL

Effects of dimensional and discrete affective word features are now well documented for adult subjects. However, we are not aware of similar studies using the ANEW or SAWL, for instance, on children. The already mentioned examples from Limbach's (2004) book and observations from daily life suggest, though, that children are already aware of emotional and even aesthetic properties of single words. We thus ran a first study using an adapted mini-version of the BAWL (*N* = 90 words compatible with text book vocabulary for age groups 7–12) on a sample of 20 children between age 7 and 12 to see to what extent the results obtained with adults could be replicated or extended (see Appendix in Supplementary Materials for Method details). The children rated these words (normally distributed on the variables valence and arousal, as taken from the BAWL06/09 databases) on valence and arousal, and additionally reported if the word was unknown or hard to imagine (imageability check). The ratings of all 20 children showed both strong valence and arousal effects and an LMM with six relevant fixed effects (valence, arousal, imageability^5^, syllables, frequency, and N) and two random effects (participants, words) showed that the standard (i.e., adult) valence and arousal values from the original BAWL were significant predictors of the children's valence ratings [t ratio (valence) = 15.37; *p* < 0.0001; t ratio (arousal) = −3.13; *p* < 0.0001], whereas only BAWL arousal was a significant predictor for the arousal ratings of the children [t ratio (arousal) = 7.36; *p* < 0.0001].

**Figure 1:**
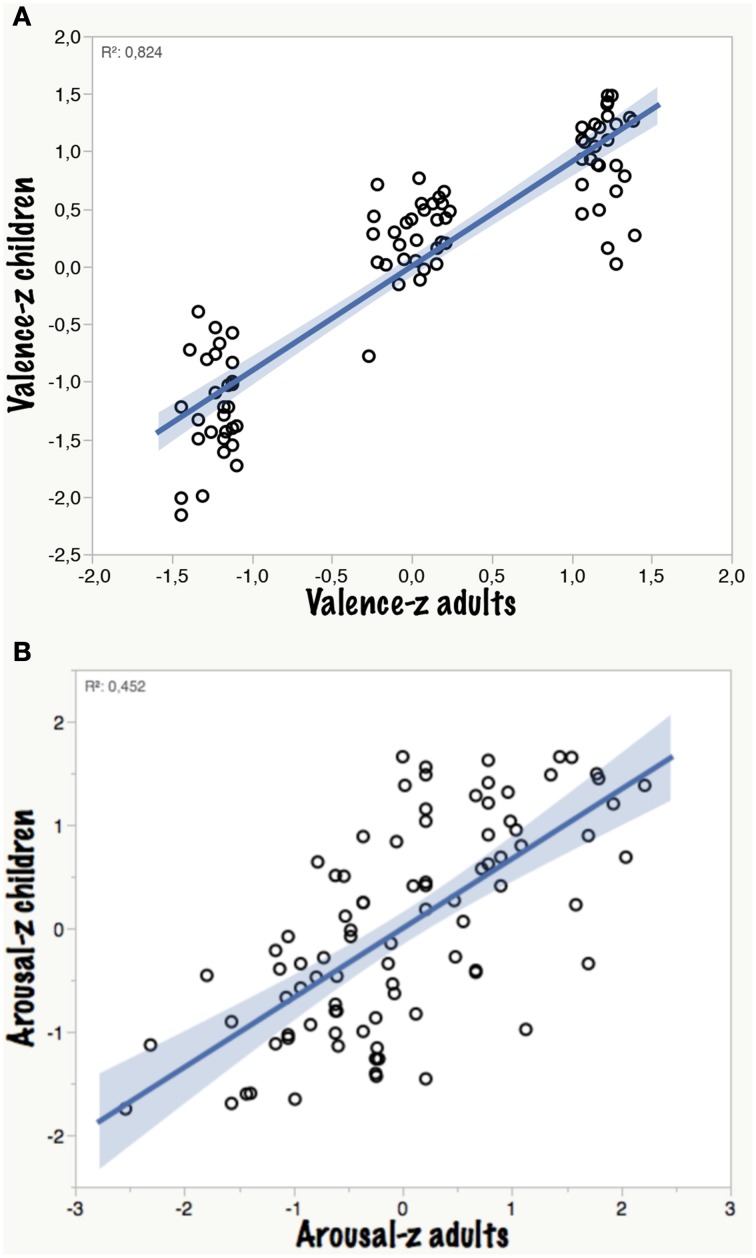
**(A,B)** Valence and arousal ratings (*z*-values) for children as a function of the original BAWL ratings (adults).

Figure 1A shows how well the adult valence ratings predict those of the children across the entire valence range: the overall correlation is high (*r* = 0.91; *p* < 0.0001) suggesting that in general at the level of categories (negative, neutral, positive) children of that age group have about the same concept of valence and/or the same judgment behavior as adults. If one breaks this down to the three valence categories, the correlations reveal a more differentiated picture: For the 30 negative words, only a quadratic correlation was significant (t ratio = −2.1; *p* < 0.045) suggesting that children use a wider range of negative ratings including extreme values, e.g., the noun GEWALT (violence) and the verb MORDEN (to kill) had more extreme *z*-values for children than for adults (−2.2 vs. −1.4 and −2 vs. −1.4, respectively). For the 30 neutral words, the linear correlation was significant (t ratio = 2.1; *p* < 0.046), whereas for the 30 positive words no significant correlation could be observed in this sample. This is due to extreme discrepancies for words like the verb KÜSSEN (to kiss) which had a much less positive *z*-value (0.3) for children than for adults (1.4). An even extremer example is the adverb OPTIMAL (optimal) with a *z*-value of 0.02 for children compared to 1.3 for adults. In contrast, the nouns MAMA (mama) or NATUR (nature) evoked more positive judgments in children (both 1.5) than in adults (both 1.2).

Figure 1B shows how well the adult arousal ratings predict those of the children: the correlation is significant but not perfect (*r* = 0.67; *p* < 0.0001; Schmidtke et al., 2014a, already document that arousal ratings generally appear to be less reliable than valence ratings). The higher intercept of the children's ratings might suggest that either they felt more aroused by the words or were more biased toward choosing higher arousal values.

Although due to the small sample size of participants and words these results might not be representative, they raise interesting questions for future studies in this underresearched field: Is there a general tendency for children to judge words associated with aggression or violence more negatively than adults? How do affective semantic fields develop over life span, and which role does age of acquisition play in this?

**Figure 2:**
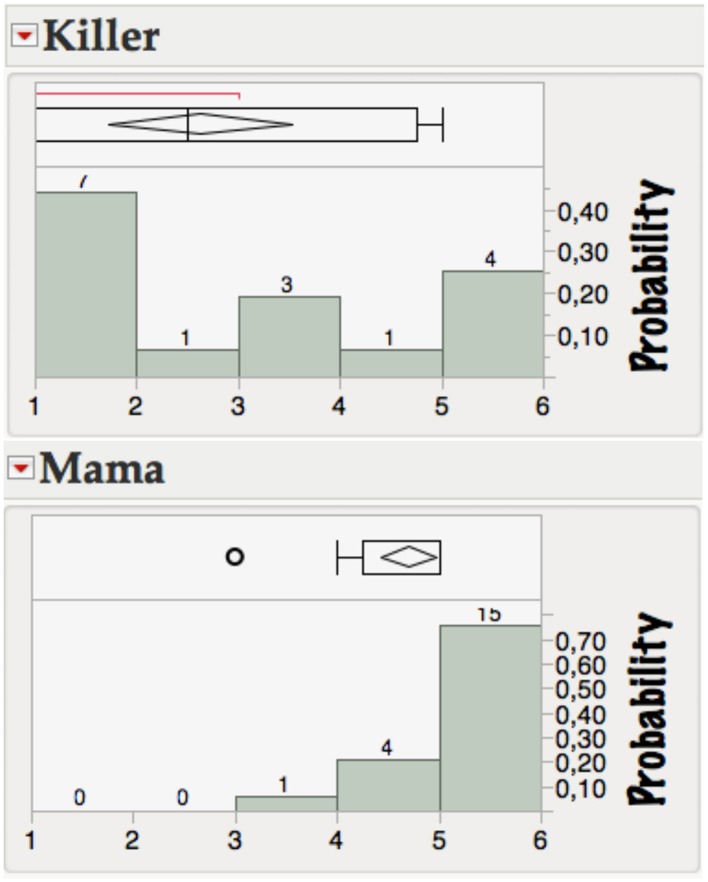
**Distribution of valence ratings (1–5) for two example words from the kidBAWL**.

To generate more research questions for future studies on affective lexical semantics, we also looked at individual items and how they differ with regard to the variation in children's valence or arousal responses. The three “least stable” words concerning valence ratings were KILLER (killer), TUMOR (tumor), and TERROR (terror) with standard deviations of ≥1.5. The three most stable were NATUR (nature), TOPFIT (topfit), and MAMA (mama) with std ≤0.5. Figure 2 shows why KILLER is affectively so ambivalent and MAMA so unambiguous: some children find KILLER very negative, but others seem to think the opposite, its *mean valence* being slightly positive (2.6/5). In contrast, the affective semantics for the word MAMA seem stable, all subjects seeing it on the “good” side of the valence scale. Although all our items were compatible with text books for children of that age group and we analyzed only words judged to be familiar, the level of comprehension for items like KILLER or OPTIMAL (see above) might, of course, still differ much more for children than for adults. This is supported by the fact that—in contrast to adults—for neutral and positive words, imageability was a significant predictor of children's valence ratings (*r*^2^ = 0.21, *p* < 0.012; *r*^2^ = 0.15, *p* < 0.035, respectively).

Our aim here is not to enter into test- or measurement theoretic issues, but to illustrate some of the complexities of trying to determine which subject- and item-related factors influence valence and arousal responses with high-dimensional word stimuli. In standard papers involving the BAWL, ANEW or similar databases, such “qualitative” analyses are not presented, but they are helpful when it comes to developing “hot” process models of reading that include affective aspects, as discussed later.

## Affectively bivalent words: the NNC-BAWL

The above KILLER example demonstrates that words can appear to have a mixed or ambivalent affective semantic structure as a result of averaging ratings across different subjects (Briesemeister et al., 2012). But can they also have an intrinsically mixed or polyvalent structure, and, if so, how valid are our valence measures? We examined this question empirically using the novel case of affectively bivalent noun-noun compounds (NNCs). One motivation for this were the results of recent computational studies using co-occurrence analyses of ultra-large databases (>10 billion words; Shaoul and Westbury, 2009; Warriner et al., 2013) to estimate the semantic structure of emotion words (Westbury et al., 2013, 2014). They demonstrated computationally that an important factor contributing to the mixed affective semantic structure of words is the “company they appear in,” thus confirming Andrews et al.'s (2009) model of lexical semantics. Using very large sample sizes, such objective co-occurrence analyses are helpful for complementing subjective rating studies, as evidenced by our recent finding that valence, arousal, or imageability judgments can be largely or entirely accounted for by two computational measures: the size and density of a word's context and the multiple emotional associations of the word (Westbury et al., 2013, 2014). Next, we present a study in which we varied the valence of the “company” of a word being part of an NNC to examine how within-word valence (in)congruities affect ratings and VDRTs.

### Uni- and bivalent NNCs

Take the word SEXBOMB and try to judge its valence and arousal. Given that the word is familiar and its processing therefore largely automatized the task is perhaps not too difficult and you will most likely rate it as positive and arousing (as average ratings suggest) despite the fact that its second component (the head) is a negative fear word. Apparently, the first word (the modifier) here is dominant for affective semantics. But what about the neologism BOMBSEX? Probably you read this word for the very first time and therefore it will take a bit longer to evaluate its emotion potential, likely due to the interactive and concurrent integration of phonological, morphosyntactic, and semantic features into a complex meaning gestalt which involves the left inferior frontal gyrus (LIFG) as a neuronal key structure (Forgacs et al., 2012). This integration process might be hindered by the feeling that the first word of the NNC is, in principle, negative, whereas the second is positive, thus creating a valence conflict which might interfere with interpreting, fluent word recognition, and the overall valence rating.

In order to examine the effects of such valence conflicts on word processing, in a recent study using the VDT we created 120 novel NNCs (10–16 letters long; see Appendix in Supplementary Materials for Method details). The NNCs were divided into four valence categories based upon the BAWL06/09 ratings for each of the two words constituting an NNC (negative valence from -3 to -1.3; positive valence from 1.3 to 3): positive-positive (PP, e.g., DUSCHVENUS/shower-venus), negative-negative (NN; PICKELHORROR/pimple-horror), positive-negative (PN; JUGENDFREITOD/youth-suicide), and negative-positive (NP; MIGRÄNEHOBBY/migrane-hobby). Participants first carried out a VDT, followed by ratings for each word on the following dimensions: valence (−3 to 3), arousal (1–5), imageability (1–7), and comprehensibility (1–7). The results of a One-Way ANOVA showed that compound type had a significant effect on VDRTs (*F* = 19.16; *p* < 0.0001), and *post-hoc t*-tests showed that both incongruous conditions had longer RTs than the congruous ones (PN: 1.95s ≤ NP: 1.98s > NN: 1.69s ≤ PP: 1.74s; all *ps* < 0.0001), but did not differ significantly from each other.

**Figure 3:**
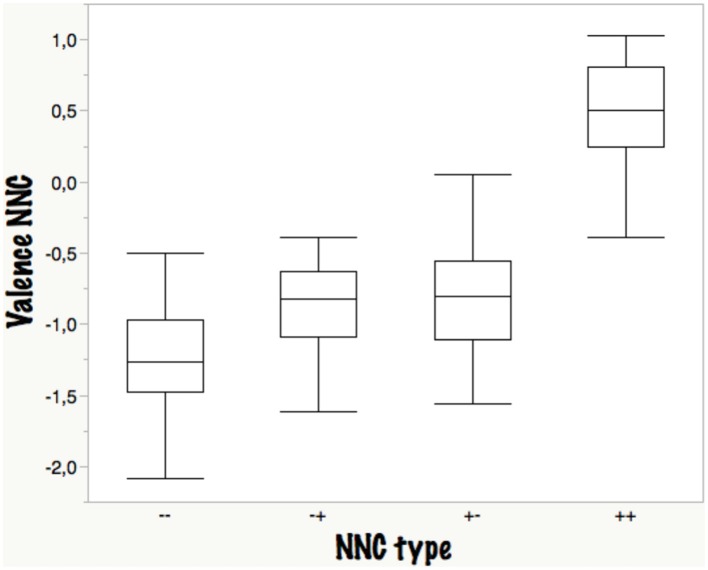
**Boxplot of valence ratings (−3 to +3) as a function of NNC type**. −−, NN; −+, NP; +−, PN; ++, PP.

Another question we asked was to what extent the rated NNC valence was determined by the valence of the two nouns (as rated independently in the BAWL06/09 studies). The box-plots in Figure 3 show that the clearest results were obtained—as could be expected—for congruous NNCs (PP, NN) with a slight advantage for double negatives, where all 30 compounds were rated as negative, whereas 4/30 PP words were rated as negative although both components were positive. The interesting result is that both incongruous NNCs had almost identical distributions and means both being rated as negative (NP: −0,85; PN: −0.83), suggesting a *negativity bias* or negative valence dominance for bivalent NNCs, independent of whether head or modifier are negative. *Comprehensibility* was ranked as follows: PP > NN ≥ PN ≥ NP, the latter three not differing significantly from each other. This finding can be explained by the fact that positive words provide a greater amount of semantic associations (Hofmann et al., 2011; Hofmann and Jacobs, 2014). Thus, semantic activation can spread across these associative pathways, and thereby elicit a *positivity bias during meaning construction*, an interesting hypothesis to be tested in future research (see also Lüdtke and Jacobs, this issue). *Arousal* was ranked: NN > NP ≥ PP ≥ NP, the only significant difference being between NN and NP.

Overall, the results indicate that valence conflicts in compounds interfere with meaning construction and raise important issues for future studies, e.g., about the time course and neuronal correlates of processing affective and other emotional or non-emotional semantic features of words (Briesemeister et al., 2014a,b). Such uni- or bivalent NNCs can be useful stimuli in studies on combinatorial semantic processing and metaphor comprehension (Forgacs et al., 2012), conflict resolution, affective word processing requiring stronger valence conditions (i.e., double-positive or –negative words), affective priming (Fazio, 2001), or cultural and existential neuroscience (Silveira et al., 2013). For example, Graupmann et al. (2013) used novel NNCs constructed from BAWL09 words (e.g., ENTENBUMERANG/duck-boomerang) as “meaning threat primes” in a recent study on cultural preferences.

## What makes words beautiful or ugly? the bBAWL

In the above mentioned book on the most beautiful German words (Limbach, 2004), the 9 year old Sylwan Wiese explains why the word LIBELLE (dragonfly) is the most beautiful for him: it has three “Ls” which is his preferred letter. This makes the word glide so well on his tongue (which is not the case for all German words). He also loves seeing them wobble and finds that the word expresses this feeling, that it ensures that one is not afraid of these insects. A deeper analysis uncovers more cues like the fact that the first four letters (LIBE-) phonologically form and perhaps unsconsciously evoke the German word for “love” (LIEBE), or that the last four (-ELLE) conjure feminine associations. Importantly, the child already mentions three cues for the beauty of words, a phonological one (the Ls), a perceptual one (the wobbling), and an affective-semantic (no fear), which supports the view that both associations with discrete emotions and embodied cognitions play a role in aesthetic appreciations of words.

The literature on word recognition and reading, however, is astonishingly mute when it comes to the issue why words can be beautiful or ugly (Schrott and Jacobs, 2011; see Bohrn et al., 2013, for an exception). In a pilot study we therefore collected 450 words from databases like the most beautiful and most ugly German words, dictionaries of German adolescent language, and the BAWL06/09 (see Appendix A5 in Supplementary Materials for Method details). Twenty subjects rated them on *valence, arousal, familiarity, imageability*, and *beauty*. Stepwise regression analyses showed that of all possible models beauty was best predicted by valence and familiarity (*r*^2^lin = 0.77; RMSE = 0.47; AICc = 608), while *arousal* and *imageability* did not account for a significant part of variance in our sample. Most interestingly, the most beautiful word in our sample was LIBELLE with a mean rating of 6.1/7, followed by MORGENRÖTE (aurora, 5.9), and MITTSOMMERNACHT (midsummernight, 5.8).

That valence predicts beauty ratings fits with the classical notion shared by scholars as different as Kant, Gadamer, or Ramashandran that pleasure is a necessary key component of aesthetic feelings (Jacobs, 2015b). That both pleasure and familiarity contribute to the subjective beauty of verbal material was also shown in a recent fMRI study by Bohrn et al. (2013) on German proverbs, which confirmed a major hypothesis of the neurocognitive poetics model of literary reading (Jacobs, 2011, 2015a,b) claiming that ancient neural systems associated with pleasure or disgust (e.g., ventral striatum; anterior insula/aINS) are involved in aesthetic feelings concerning verbal material, i.e., the Panksepp–Jakobson hypothesis mentioned in the introduction. Concerning the backside of beauty, i.e., ugliness, another recent study combining intracranial and surface EEG also confirmed this hypothesis by showing that as early as 200 ms post-stimulus the aINS significantly responded to disgusting words (Ponz et al., 2013). Such results challenge standard “cold cognitive” models of word recognition and reading, which so far ignore affective features of words and do not include subcortical or limbic structures in the “reading network” (cf. Hofmann and Jacobs, 2014).

To obtain an idea about which semantic features contribute to the beauty or ugliness of words, we ran a hierarchical cluster analysis over the five rated variables yielding an adequate set of 13 clusters (cubic clustering criterion = 2.5). Table A1 (Appendix in Supplementary Materials) gives 10 example words of the extreme clusters 1 and 12. The most beautiful words of Cluster 1 overall described nine phenomena from nature (animals, flowers, rainbow etc.) and four states/objects of wellness (e.g., coziness), all rated high on beauty, valence, and imageability, and low on arousal. In contrast, the overall 24 “ugliest” words from cluster 12 were almost all swear words associated with genitalia.

Naturally, our pilot study on the beauty of words is only a beginning. The above re-analysis of the BAWL06 data as well as the intuitive evidence by the contributors to the book “The most beautiful German word” suggest that associations with dimensional and discrete emotions, as well as embodied features also contribute to beauty ratings, as probably do sublexical factors such as phoneme valence (Aryani et al., 2013, in press), and phonological iconicity (Schmidtke et al., 2014b), or lexical ones like the sound image of words (Ullrich et al., this issue).

## Toward a neurocomputational model of the VDT and affective word recognition

The above results from various studies using the BAWL as a tool for revealing aspects of the processing of affective words have shed light on different factors affecting valence or lexical decisions in adults and children with simple or complex words. They can thus motivate and constrain the development of “hot” process models of word recognition, sentence comprehension, or text processing that would include affective and aesthetic processes (Jacobs, 2015a,b). In the following, we would like to discuss some elementary features of such a model starting with computational aspects and ending with three tentative neurocognitive models. The aim of these models is to help answer the question *How* exactly subjects go about when judging the valence of high-dimensional “symbolic” stimuli like words, as in the VDT. Related questions to be answered by any neurocomputational process model concern the *Where* (functional, neuroanatomical) and *When* of the effects observed with affective words (cf. Kissler et al., 2006; Citron, 2012; Hofmann and Jacobs, 2014). While the behavioral data presented in this paper do not directly speak to the latter two questions, the studies using BAWL stimuli from our lab and others, summarized in Table 1, do so and thus—together with other literature—provide a basis for the following theoretical considerations.

Perhaps the most basic information provided by the above studies with regard to such modeling projects is that already seven to 12 year old children show a well developed ability to judge the valence and arousal of words indicating that they have access to their affective semantic features. Whether these are the result of contextual learning/evaluative conditioning processes (Fritsch and Kuchinke, 2013), or some other unknown mechanism linking emotional and embodied experiences to words is still an open question, though. It seems safe to assume, however, that the processes determining valence and arousal values are triggered by some visual and/or linguistic features of a word which are perceived before a valence decision takes place. In principle, these features could be of sublexical or lexical origin, or both, and—if we assume automatic phonological recoding of written words and multiple embodied associations (Bühler, 1934; Jacobs et al., 1998; Yap et al., 2012)—they can be visual, phonological, multi-sensory-motor, or some combination. If in analogy to the model by Andrews et al. (2009) the affective meaning of words is best understood as the result of learning the statistical structure underlying a single joint distribution of both experiential and distributional data, then valence and arousal could be seen as *semantic supra-features* that result from (i) neural activation patterns distributed over the sensory-motor representations of their referents (experiential aspect) and (ii) the linguistic company the words keep, i.e., the size and density of their context, as computationally modeled using co-occurrence statistics (Hofmann and Jacobs, 2014).

The second message of the present empirical results and previous literature for model construction is that the experiential aspect would include both associations with discrete emotions and embodied features, whereas the distributional aspect would include partial or full transfer of the valence and arousal features of the context words to the target word via affective spreading activation (Hofmann and Jacobs, 2014). The distributional aspects would hypothetically contribute less strongly to the arousal value than the experiential ones, if arousal is considered the more direct and body-related variable of the two. A recent computational study by Westbury et al. (2014) indeed suggests that arousal ratings are associated more strongly with autonomic reactivity than valence, predicted by co-occurrence similarity to emotion labels naming automatic emotional reactions (e.g., the words HUMILIATION, LUST, and PANIC). In contrast, the best computational model of valence ratings was very different, and had a clear structure suggesting that they are highly associated with four dimensions: *potency (strong-weak), happiness, approachability (bad-pleasure)*, and *anger/rage*.

A third message of our results is that models of affective word processing should take into account the mixed affective semantic structure (or ambi- and polyvalence) of many words, whether for simple nouns like KILLER or for more complex NNCs like SEXBOMB. This calls for a change in methodology when studying affective word recognition, using both a bipolar and a bivariate approach to see which one provides a better fit to the data (Briesemeister et al., 2012). A final dispatch of the present data for future models of affective and aesthetic word recognition is that valence and familiarity likely play a greater role than arousal and imageability for the judged beauty of words.

### Computational models of affective word recognition

Computational models of affective word recognition must specify *Where* and *When* in the model the factors arousal, valence, or semantic associations exert their influence and *How* these factors interact in determining a valence or lexical decision. Given the success of interactive activation models (IAMs) in predicting “cold” word recognition performance in the LDT and in making the underlying processes transparent, i.e., algorithmically concrete (e.g., Grainger and Jacobs, 1996; Hofmann and Jacobs, 2014), they also are a good candidate for simulating “hot” affective word processing in the VDT or LDT. First steps in this direction were made with the models of Siegle et al. (2002) and Kuchinke (2007) whose MROMe (Multiple Read-Out Model emotional) could account for faster lexical decisions in positive words (Kuchinke et al., 2005, 2007) by an evaluation mechanism added to the original MROM (Grainger and Jacobs, 1996). However, it could not predict RT differences between positive and negative words, such as the RT advantage for positive words found in Võ et al. (2006), or Briesemeister et al. (2011a,b). A further development trying to overcome limitations of previous models is the Associative Read-Out Model (AROM; Hofmann et al., 2011) which extends the scope of IAMs by introducing explicit memory and semantic representations necessary for implementing emotional aspects. Using this model, Hofmann and Jacobs (2014) presented evidence suggesting that positive valence effects can be explained by semantic cohesion (i.e., the higher semantic-associative cohesiveness of affective words compared to neutral ones), as suggested by Phelphs et al. (1998). A future neurocomputational model trying to account for the *How* of affective and aesthetic word recognition should augment the aforementioned models with clear predictions regarding the *Where* and *When*, i.e., the neurofunctional/-anatomical locus and time-course of valence (or other affective) effects. However, the time for a unified model does not seem to be ripe yet, since currently this could be done from at least three different theoretical perspectives which are sketched in the following section.

### Bipolar perspective

Regarding the neurofunctional *Where* question, from a first perspective viewing valence as a *bipolar* construct, Amy and aIns activations—or an amygdalar-hippocampal network (Kensinger and Corkin, 2004)—are primarily associated with arousal, whereas valence is most often associated with OFC (Lewis et al., 2007), ventral anterior (as well as posterior and subgenual) cingulate cortex (vACC; Maddock et al., 2003), inferior frontal (Briesemeister et al., 2014b) or a prefrontal cortex-amygdalar network (Kensinger and Corkin, 2004; Schlochtermeier et al., 2013). When looking at the *When* question, i.e., temporal word recognition ERP effects of valence and arousal (see Citron, 2012; or Kissler et al., 2006, for reviews) there is evidence that arousal comes first (N1; Hofmann et al., 2009; Kissler and Herbert, 2012), followed by valence (early posterior negativity/EPN, late positivity complex/LPC; Recio et al., 2014), with reward being in between (P2; Schacht et al., 2012). But there is also data suggesting that valence comes first (P1; e.g., Bayer et al., 2012a), followed by arousal (EPN; Bayer et al., 2012b). All these results were obtained with BAWL or ANEW-type words, but appear somewhat inconsistent, sometimes even from within the same lab (e.g., Bayer et al., 2010, 2011; Palazova et al., 2011; Rellecke et al., 2011): some data suggest very early “pre-lexical” effects of valence (P1), others late, “post-lexical” effects (LPC)^6^. It also remains unclear to what extent valence and arousal effects on ERPs interact (Citron et al., 2014; Recio et al., 2014), or are confounded with effects of discrete emotional information like joy or disgust (N1; Briesemeister et al., 2014a,b; EPN; Ponz et al., 2013). At the behavioral and peripheral-physiological levels, word valence influences RTs and ratings, presumably triggered by discrete emotion and/or embodied features, and albeit to a much lesser and more uncertain degree also autonomous nervous system variables, like corrugator, electrodermal, and pupillary activity (Võ et al., 2008; Bayer et al., 2011, but see Kuchinke et al., 2007 for null findings in LDT). However, much as for neuroimaging data, results are inconsistent, sometimes showing shorter RTs for positive words, sometimes for negatives, sometimes no advantage compared to neutral words (Hofmann and Jacobs, 2014).

### Bivariate perspective

A second perspective views valence as a *bivariate* construct (e.g., Norris et al., 2010; Briesemeister et al., 2012), relating it to notions of reward and behavioral activation (positivity) vs. punishment and behavioral inhibiton (negativity). In this perspective, *positivity* is neuroanatomically most often associated with the basal ganglia (BG) including the ventral striatum (VS), left frontal pole (lFP), mOFC, vmPFC, pCC, and SMA, whereas *negativity* is rather associated with insula, right amygdala (rAmy), PAG, rdACC, lOFC, dmPFC, and deep cerebellar areas (Maddock et al., 2003). We are not aware of studies answering the *When* question of positivity vs. negativity activation, but Norris et al. (2010) summarize behavioral, peripheral-physiological, and ERP research supporting the negativity bias and positivity offset hypotheses of this perspective and thus providing indirect evidence for it.

### Interactive perspective

Finally, a third theoretical perspective merits discussion, because some results suggest that valence and arousal affect processing of emotional stimuli in an interactive way (Herbert and Kissler, 2010; Citron et al., 2014). According to this perspective, stimuli with negative valence (e.g., bitter taste) or high arousal (e.g., a loud noise) elicit a *withdrawal* tendency and corresponding mental set, because they represent a possible threat. In contrast, stimuli with positive valence (e.g., sweets) or with low arousal (e.g., a newsletter) elicit an *approach* tendency because they are perceived as safe (Briesemeister et al., 2013). These two tendencies are hypothesized to be initiated independently at a pre-attentive level and subsequently integrated in order to evaluate the stimulus for further action. This perspective predicts that positive low-arousal and negative high-arousal stimuli (are easier to process, because they elicit congruent tendencies (approach and withdrawal, respectively), whereas positive high-arousal and negative low-arousal stimuli are more difficult to process because they elicit conflicting approach-withdrawal tendencies. At the neurofunctional level, Citron et al. (2014) recently reported evidence for this perspective showing greater neural activation within right insular cortex in response to stimuli evoking conflicting approach-withdrawal tendencies (i.e., positive high-arousal and negative low-arousal words; PosHi; NegLo) compared to stimuli evoking congruent approach vs. withdrawal tendencies (i.e., positive low-arousal and negative high-arousal words; PosLo; NegHi). Further supporting evidence comes from ERP studies in favor of the approach-withdrawal assumption and the idea of the emotional and motivational embodiment of words (Herbert and Kissler, 2010; Herbert et al., 2012, 2013).

**Figure 4:**
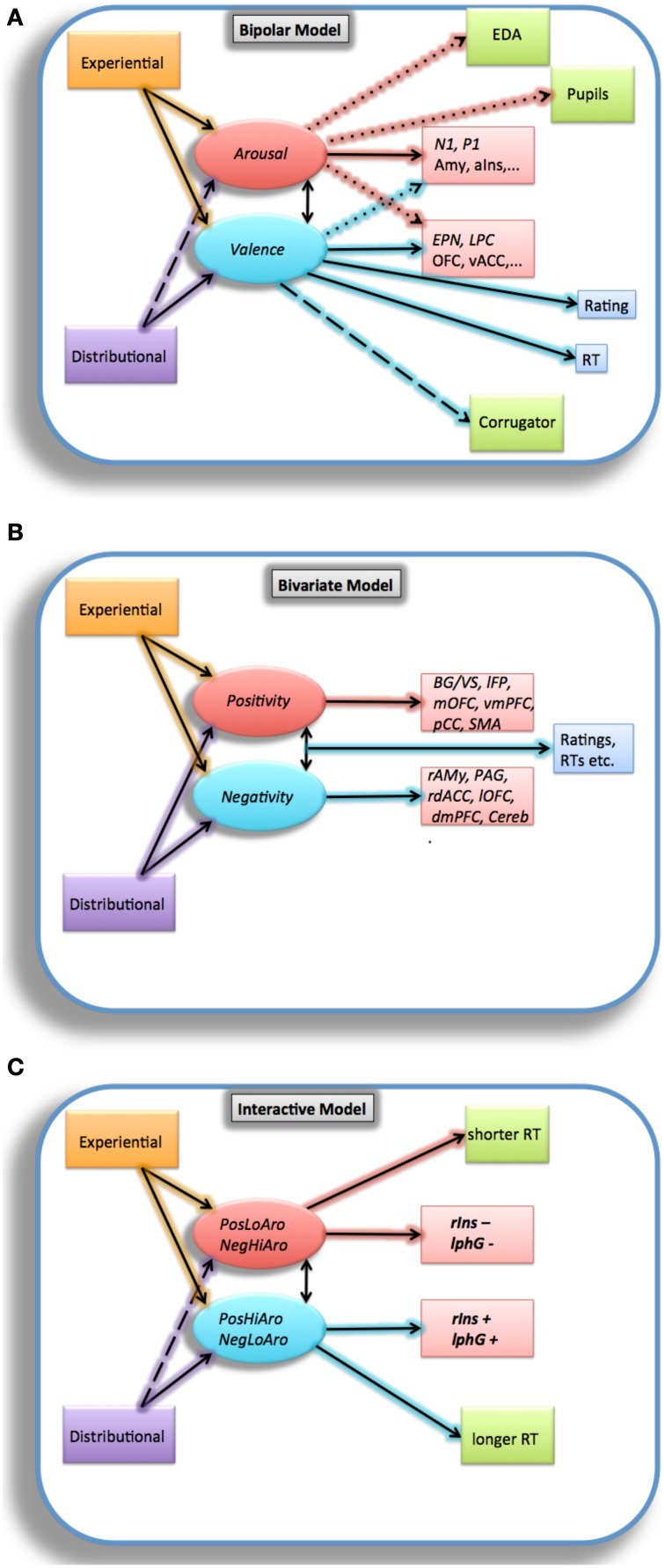
**(A–C)** Diagrams showing hypothetical relations between affective word variables and their effects at the behavioral, brain-electrical, and neurofunctional levels. Continuous-line arrows assume strong relations, interrupted and dotted lines weaker, more questioneable ones. Abbreviations: **(A)** EDA, Electrodermal activity; Amy, amygdala; aIns, anterior insula; EPN, early posterior negativity; LPC, late posterior complex; OFC, orbitofrontal cortex; vACC; ventral anterior cingulate cortex. **(B)** BG, basal ganglia; VS, ventral striatum; lFP, left frontal pole; mOFC, medial orbitofrontal cortex; vmPFC, ventromedial prefrontal cortex; pCC, posterior cingulate cortex; SMA, supplementary motor area; rAMy, right amygdala; PAG, periaqueductal gray; rdACC, right dorsal anterior cingulate cortex; lOFC, left orbitofrontal cortex; dmPFC, dorsomedial prefrontal cortex; Cereb, cerebellum. **(C)** PosLoAro, positive valence, low arousal; NegHiAro, negative valence, high arousal; PosHiAro, positive valence, high arousal; NegLoAro, negative valence, low arousal; rIns, right Insula; lphG, left parahippocampal gyrus.

These considerations are sketched in the hypothetical diagrams of Figure 4. Figure 4A sketches the bipolar model of valence. Figure 4B sketches the bivariate interpretation of valence, arousal being left out, because it plays no key role in this perspective, which also makes no specific hypotheses with regard to differential effects of bivariate valence on RTs or ratings^7^ (Norris et al., 2010). Finally, Figure 4C sketches the interactive view. Note that all models incorporate the view that valence and arousal are affective super-features derived from experiential and/or distributional word properties including discrete and embodied features processed during an earlier phase (Briesemeister et al., 2014a,b).

## Conclusion

The present paper offers an overview about the lessons we learned from previous versions of the BAWL, and discusses some future perspectives characterizing the affective connotation of words on embodied, developmental, discrete-emotion, and aesthetic dimensions of meaning. This enriched perspective on word processing is further complemented by analyses based on the co-occurrence of words that either reduce the dimensions of meaning or explain positivity by semantic processes. These approaches provide a first step toward neuro-computationally concrete models of affective word, sentence, and text processing, which we see as the major challenge for the future (Jacobs, 2015a,b).

### Conflict of interest statement

The authors declare that the research was conducted in the absence of any commercial or financial relationships that could be construed as a potential conflict of interest.

## Footnotes

^1^Implicit with regard to the affective processing of words.

^2^Initial analyses showed that using logRT instead of RT improved model fits significantly (cf. Janssen, 2012).

^3^In order to keep the analyses simple given the total number of variables, and since logRT transforms can have nonlinear effects on interactions (Kliegl et al., 2010), we did not introduce any interaction terms into the present LMM analyses. Future studies should look into theoretically and empirically well founded interactions between the 20 variables examined here (cf. Yap et al., 2012).

^4^The thumb rule is that a difference of 10 points is usually significant (Janssen, 2012).

^5^Adult ratings taken from the BAWL06/09 studies.

^6^The notions pre- and post-lexical can easily be interpreted in the framework of serial stage models of word recognition, but require a more differentiated definition when using nonlinear dynamic processing models from e.g., the IAM family. This is because in IAM-type models, the lexical feedback loop changes activation at the sub- or prelexical levels (e.g., letters, syllables). Thus, in this model context “prelexical” would mean a pure bottom-up effect, not affected by any lexical feedback. Since lexical feedback typically requires four to seven processing cycles to show sublexical effects, any “prelexical” effect predicted from such models must occur extremely early.

^7^With the exception of mood ratings.
