# Supplementary material for: 10 years of BAWLing into affective and aesthetic processes in reading: what are the echoes?
Source: Front Psychol. 2015 Jun 3;6:714. doi: 10.3389/fpsyg.2015.00714 (PMC4452804; doi:10.3389/fpsyg.2015.00714)
Supplement: Supplementary file 1 [file DataSheet1.DOCX]

**Appendix**

**A1. Methods for eBAWL study**

**Participants**. N = 104 (82 female; age: M= 29.25, SD=7.63, range=19-51) subjects, all native German speakers gave written consent to participate in the study, which – like all other studies reported here - was conducted in accordance with the regulations of the Free University’s Research Ethics Board.

**Material, design, procedure**. 689 words across all word categories (nouns, adjectives and verbs) were selected from the BAWL, subdivided into five lists containing 110 items and into one list containing 139 items. Ratings were collected via an Internet-based html script running on a public server provided by the Free University Berlin. Participants were instructed to carefully read the presented word and then indicate on five independent 5-point Likert scales the intensity of the elicited sensation of ‘seeing’, ‘hearing’, etc. the object/event/action denotated by the word (1 = low intensity, 5= strong intensity). Each word was presented individually in black uppercase letters (font type Times New Roman, font size 18 pt) on a white background. Participants were able to individually decide when to advance to the next trial by clicking on a button. Word order was randomized for each subject. Each participant rated only one of the six different lists. Online ratings were then averaged offline per item and per rating. Each word received ratings from at least 15 different participants.

**A2. LMM analyses**

The LMMs were computed using the JMP-Pro 11 (SAS Institute Inc., Cary, NC) software package’s ‚mixed model’ routine. We did not include maximal random slope adjustments (as in Lüdtke & Jacobs, this volume), since no within- or between-subject categorical variables were used in our design, and use of maximal random-effects structures with our many continuous variables (14) would have resulted in an uneven pattern of model nonconvergences (cf. Whitford & Titone, 2014). The stepwise approach seems justified since our present approach is not a designed experiment but a post-hoc analysis with many continuous variables and an exploratory rather than a conclusive character. The fit statistics, random effects covariance, and fixed effects parameter estimates for the 14V model are given below in Table A1a-c.

**Table A1a-c.**

**a) Fit Statistics**

-2 Residual Log Likelihood 7030,4355

-2 Log Likelihood 6955,6347

AICc 6991,8607

BIC 7100,0173

**b) Random Effects Covariance Parameter Estimates**

Covariance Parameter Estimate Std Error 95% Lower 95% Upper

PARTICIPANT 0,3811758 0,1217816 0,1424882 0,6198633

WORD 0,0436788 0,0085573 0,0269067 0,0604508

Residual 0,5333841 0,0140662 0,5068539 0,5620637

**c) Fixed Effects Parameter Estimates**

Term Estimate Std Error DFDen t Ratio Prob>|t| 95% Lower/95% Upper

Intercept 1,3165135 0,2708712 136,0 4,86 <,0001 0,7808497/1,8521773

Joy -0,269458 0,0612382 134,3 -4,40 <,0001 -0,390574/0,148342

Anger -0,1583 0,0495598 134,4 -3,19 0,0017 -0,256318/0,060282

Sadness -0,13893 0,0657043 136,0 -2,11 0,0363 -0,268864/0,008996

Fear 0,0542299 0,0744576 134,5 0,73 0,4677 -0,093020/0,2014892

Disgust -0,007061 0,0580383 132,8 -0,12 0,9034 -0,12186/0,107738

Valence -0,015966 0,0560404 134,0 -0,28 0,7762 -0,126804/0,0948722

Arousal -0,135349 0,0508321 133,4 -2,66 0,0087 -0,235889/0,034808

Imageability -0,027548 0,0241096 134,0 -1,14 0,2552 -0,075233/0,0201366

Phonemes 0,0394018 0,0213202 133,8 1,85 0,0668 -0,002766/0,08157

logF -0,027007 0,0150678 134,0 -1,79 0,0753 -0,056808/0,0027948

N 0,0011462 0,0176126 134,6 0,07 0,9482 -0,033687/0,0359793

TASTE -0,026117 0,0264839 133,0 -0,99 0,3259 -0,078501/0,0262674

GRASP 0,0294578 0,0298359 134,6 0,99 0,3253 -0,02955/0,0884655

MOVE 0,1010271 0,0311459 133,8 3,24 0,0015 0,0394252/0,162629

**A3. Methods for kidBAWL study**

**Participants**. 20 children (age M = 9.2, SD = 1.4), all native German speakers, took part in the study (verbal consent of the children and written consent of the parents was obtained).

**Material, design, procedure**. The kidBAWL is a list of 2045 words selected by Sylvester (2013) from the BAWL-R, matched with a dictionary for children of that age range (Sennlaub, 2007; 2012). The stimulus material consisted of 90 words taken from the kidBAWL: 30 positive, 30 neutral, and 30 negative ones. Words were presented on a laptop using the Psychopy software (Peirce, 2007) in type font times new roman (size 40; 1.3 cm) on top of either a row of smileys for valence ratings, a row of self-assessment manikins for arousal ratings (SAM, Lang, 1980), or a row of boxes for imageability ratings. Each session started with a short questionnaire, a standardized instruction, and three training trials. Each trial started with a familiarity rating of the word. Unfamiliar words were discarded from the analyses. Trials were self-paced and participants could interrupt the session any time, e.g. to ask the experimenter questions. Sessions lasted between 18 and 42 minutes. Participants were rewarded afterwards by a pack of gummy bears.

**A4. Methods for NNC-BAWL study**

**Participants**. 36 students of FUB (28 female, 8 male), all native German speakers, took part in the experiment for course credit.

**Material, design, procedure**. 120 NNC stimuli were created from words (5 – 8 letters long) of the BAWL-R having valence values of -3 to -1.3, and 1.3 to 3.0, respectively. The BAWL-R words, matched for mean frequency, mean imageability, and SD of valence, were combined to form the four types of novel NNCs (PP, NN ,PN, NP). Stimuli were presented on PCs for 500 ms using the Psychopy software (Peirce, 2007) in type font times new roman (size 20; white on black background). Subjects had to press response keys to decide as quickly and accurately as possible whether the stimulus was negative or positive. After the VDT, subject rated all stimuli with regard to valence, arousal, imageability, and comprehensibility.

**A5. Methods for bABWL study**

**Participants**. 20 students of FUB (14 female, 6 male), all native German speakers, took part in the study for course credit.

**Material, design, procedure**. A total of 450 words were collected from databases like the most beautiful and most ugly German words, dictionaries of German adolescent language, and the BAWL06/09. Stimuli were presented on PCs using the Psychopy software (Peirce, 2007) in type font times new roman (size 20; white on black background). Subjects rated all stimuli with regard to valence, arousal, imageability, and beauty.

**TABLE A2. Examples from cluster analysis on 450 beautiful and ugly German words with average ratings (1 – 7) for:** Valence = V, Imageability = I, Familiarity = F, Beauty = B, and Arousal = A.

| **Cluster 1** | **V** | **I** | **F** | **B** | **A** |
| --- | --- | --- | --- | --- | --- |
| Libelle [dragonfly] | 5.57 | 6.86 | 6.64 | 6.07 | 2.29 |
| Regenbogen [rainbow] | 6.36 | 6.93 | 6.57 | 5.86 | 1.86 |
| Schäfchenwolken [fluffy clouds] | 6.43 | 6.86 | 6.43 | 5.86 | 1.43 |
| Tautropfen [dewdrop] | 6.07 | 6.64 | 6.29 | 5.86 | 1.64 |
| Mondschein [moonshine] | 6.43 | 6.86 | 6.71 | 5.79 | 1.93 |
| Blütenstaub [blossom dust] | 5.64 | 6.29 | 6.21 | 5.71 | 1.57 |
| Zitronenfalter [brinstone butterfly] | 5.93 | 6.57 | 6.36 | 5.64 | 2.14 |
| Weidenkätzchen [pussy willow] | 6.14 | 6.36 | 5.93 | 5.57 | 1.57 |
| Gemütlichkeit [coziness] | 6.29 | 6.36 | 6.64 | 5.50 | 1.57 |
| Behutsamkeit [gingerliness] | 6.00 | 5.57 | 6.29 | 5.21 | 1.36 |
| **Cluster 12** |  |  |  |  |  |
| Arschgeburt [arse birth] | 1.75 | 3.88 | 3.71 | 1.79 | 3.04 |
| Bettpisser [bed-pisser] | 1.79 | 4.50 | 3.79 | 1.79 | 2.71 |
| Fickfehler [fuck-mistake] | 2.29 | 2.38 | 1.88 | 1.79 | 2.67 |
| Kotzbrocken [son of a bitch] | 2.00 | 4.13 | 5.04 | 1.71 | 2.42 |
| Pickel [pimple] | 2.08 | 5.96 | 5.92 | 1.67 | 2.63 |
| Afterlecker [arse licker ] | 1.54 | 3.75 | 1.88 | 1.63 | 2.83 |
| Kotzen [to puke] | 1.50 | 5.58 | 5.71 | 1.63 | 2.88 |
| Fotzenkäse [cunt cheese] | 1.63 | 2.63 | 1.88 | 1.54 | 3.04 |
| Gesichtsfotze [cunt face] | 1.67 | 3.21 | 2.79 | 1.54 | 2.71 |
| Fotze  [twat] | 1.71 | 4.63 | 5.33 | 1.42 | 3.17 |
